# L-mimosine and hypoxia can increase angiogenin production in dental pulp-derived cells

**DOI:** 10.1186/s12903-017-0373-6

**Published:** 2017-05-25

**Authors:** Klara Janjić, Michael Edelmayer, Andreas Moritz, Hermann Agis

**Affiliations:** 1 Department of Conservative Dentistry and Periodontology, School of Dentistry, Medical University of Vienna, Sensengasse 2a, 1090 Vienna, Austria; 2 Austrian Cluster for Tissue Regeneration, Donaueschingenstr. 13, 1200 Vienna, Austria; 3 Department of Oral Surgery, School of Dentistry, Medical University of Vienna, Sensengasse 2a, 1090 Vienna, Austria

**Keywords:** Angiogenin, ANG, Dental pulp, Prolyl hydroxylases, Prolyl hydroxylase inhibitors, Regeneration, Hypoxia, Hypoxia mimetic agents, Microtissues, Echinomycin

## Abstract

**Background:**

Angiogenin is a key molecule in the healing process which has been successfully applied in the field of regenerative medicine. The role of angiogenin in dental pulp regeneration is unclear. Here we aimed to reveal the impact of the hypoxia mimetic agent L-mimosine (L-MIM) and hypoxia on angiogenin in the dental pulp.

**Methods:**

Human dental pulp-derived cells (DPC) were cultured in monolayer and spheroid cultures and treated with L-MIM or hypoxia. In addition, tooth slice organ cultures were applied to mimic the pulp-dentin complex. We measured angiogenin mRNA and protein levels using qPCR and ELISA, respectively. Inhibitor studies with echinomycin were performed to reveal the role of hypoxia-inducible factor (HIF)-1 signaling.

**Results:**

Both, L-MIM and hypoxia increased the production of angiogenin at the protein level in monolayer cultures of DPC, while the increase at the mRNA level did not reach the level of significance. The increase of angiogenin in response to treatment with L-MIM or hypoxia was reduced by echinomycin. In spheroid cultures, L-MIM increased angiogenin at protein levels while the effect of hypoxia was not significant. Angiogenin was also expressed and released in tooth slice organ cultures under normoxic and hypoxic conditions and in the presence of L-MIM.

**Conclusions:**

L-MIM and hypoxia modulate production of angiogenin via HIF-1 differentially and the response depends on the culture model. Given the role of angiogenin in regeneration the here presented results are of high relevance for pre-conditioning approaches for cell therapy and tissue engineering in the field of regenerative endodontics.

## Background

Angiogenin (*Angiogenin* / ANGIOGENIN) is a key molecule in the healing process which has been successfully applied in the field of regenerative medicine and oncology [1–7]. The applications comprise the support of wound healing and bone regeneration where it has successfully stimulated angiogenesis and tissue regeneration in experimental settings [3, 4, 8]. While the known relevance of angiogenin is based on a broad spectrum of data in regenerative medicine, knowledge about the role of angiogenin in regenerative endodontics and the dental pulp in general is currently limited. In vitro, cells of the dental pulp produce *angiogenin* / ANGIOGENIN and fast-setting calcium-silicate cements can modulate the production of *angiogenin* / ANGIOGENIN [9, 10]. In inflammation, innate immunity, neuroprotection, and tissue regeneration ANGIOGENIN represents a factor with high relevance also for regenerative endodontics [11–14].

ANGIOGENIN is a secreted protein and part of the ribonuclease superfamily for which reason it is also known as ribonuclease 5 [8]. The ANGIOGENIN encoding gene *angiogenin* is found on chromosome 14q11 [15]. The 14kDa basic single-chain protein consists of 123 amino acids [8] and has 33% sequence identity and 65% homology with bovine pancreatic RNase A as well as the same general catalytic residues [16]. The structure of the ANGIOGENIN protein is characterized by two α-helices, seven β-sheets, and three disulfide bonds [8].

*Angiogenin* / ANGIOGENIN is found in the early phase during hard and soft tissue regeneration [17, 18]. ANGIOGENIN is involved in angiogenesis and thus a key factor in cancer genesis but also tissue regeneration [1–7]. Various pro-angiogenic factors such as vascular endothelial growth factor, fibroblast growth factor, and endothelial growth factor stimulate *angiogenin* / ANGIOGENIN production [8, 19]. In the complex process of angiogenesis, ANGIOGENIN induces a variety of cell responses. ANGIOGENIN binds to endothelial and smooth muscle cells triggering cell migration, cell invasion, proliferation of endothelial cells, and formation of tubular structures [19, 20]. These functions are based on ANGIOGENIN’s ribonuclease activity, basement membrane degradation, signaling transduction, and nuclear translocation [21, 22]. Furthermore, emerging evidence supports the notion that ANGIOGENIN is involved in the cell response to stress and cell survival [23]. All these processes highlight the relevance of ANGIOGENIN and led to the development of therapeutic strategies which target this mechanism [3, 4].

Hypoxia-based strategies are a novel and promising approach to stimulate healing of the dental pulp in regenerative endodontics. While this approach emerged in a wave of various hypoxia-based approaches in regenerative medicine which dramatically improved our understanding of the effects of hypoxia, the impact of hypoxia in the dental pulp is not fully understood [24–30]. There is exisiting evidence from other fields of research that hypoxia can induce *angiogenin* / ANGIOGENIN via the transcription factor hypoxia-inducible factor (HIF)-1 [31, 32]. The role of ANGIOGENIN in the dental pulp is, however, unknown.

Here we hypothesized that hypoxia stimulates the production of *angiogenin* / ANGIOGENIN in the pulp in 2D monolayer, 3D spheroid, and tooth slice organ cultures. We show that the hypoxia mimetic agent L-mimosine (L-MIM) and hypoxia modulate production of *angiogenin* / ANGIOGENIN via HIF-1 differentially and the response depends on the culture model. These results are of high relevance for pre-conditioning approaches for cell therapy and tissue engineering in the field of regenerative endodontics which aim to improve the pro-angiogenic capacity of the transplanted cells.

## Methods

### Cell culture

Human dental pulp-derived cells (DPC) were prepared from extracted third molars after informed consent was given by the donors (Ethics Committee of the Medical University of Vienna, Vienna, Austria). Patients were recruited at the School of Dentistry, Medical University of Vienna, Vienna, Austria. The extraction of third molars from donors was part of standard care. The dental pulp was exposed and the tissue was collected. Explant cultures were done in α-minimal essential medium (α-MEM) (Invitrogen Corporation, Carlsbad, CA, USA) with 10% fetal calf serum (FCS; PAA Laboratories, Linz, Upper Austria, Austria) and antibiotics at 37 °C, 5% CO_2_, and 95% atmospheric moisture.

### Monolayer culture of dental-pulp-derived cells

For the monolayer cultures, DPC were seeded at 50,000 cells/cm^2^ and incubated overnight. Then DPC were treated with 1 mM L-MIM or hypoxia for 24 h. This dose was based on the results of previous in vitro studies [24, 33]. For hypoxia, an established assay was applied with minor modifications [34]. In brief, DPC were placed into a BD GasPak EZ Pouch system for hypoxic conditions (Becton, Dickinson and Company, Franklin Lakes, NJ, USA). In indicated experiments, cells were also treated with echinomycin at 1 μM to block HIF-1 activity. Untreated cells cultured under normoxic conditions served as control.

### Spheroid culture of dental pulp-derived cells

For the spheroid cultures, 3D Petri Dishes® (Microtissues Inc., Providence, RI, USA) were used. The dishes were filled with 2% agarose to produce molds with 35 circular recesses. The molds were then soaked in α-MEM supplemented with 10% FCS and antibiotics (Invitrogen Corporation, Carlsbad, CA, USA). Afterwards, the molds were transferred to the well plates and 75 μL of cell suspension with 7,300,000 cells/mL were pipetted into the molds. After 10 min settling time the well was filled with cell culture medium as described in the manufacturer’s description and incubated overnight. Then spheroids of DPC were stimulated with L-MIM or hypoxia as described above in the monolayer cultures.

### Tooth slice organ culture

After informed consent was obtained, 600 μm thick slices from extracted third molars without any sign of inflammation were prepared. For slicing the teeth, a diamond saw (Exakt 300 CL and D64 0,2 mm, EXAKT Norderstedt, Germany) was used. Directly after cutting, the slices were placed in α-MEM (Invitrogen Corporation) supplemented with 10% FCS and antibiotics for 48 h at 37 °C, 5% CO_2_, and 95% atmospheric moisture. Then the tooth slices were treated with L-MIM at 1 mM or hypoxia for 48 h according to the concentrations used in previous studies [24, 35]. From one set of tooth slices total RNA was extracted and *angiogenin* mRNA levels were assessed using RT-qPCR. Culture supernatants were assessed by ELISA to quantify the amount of ANGIOGENIN protein. Furthermore, MTT assays were applied on the tooth slices to quantify the vital tissue. In brief, tooth slices were incubated with 3-(4,5-dimethylthiazol-2-yl)-2,5-diphenyltetrazolium bromide (MTT) at 1 mg/mL for 2 h. Formazan was solubilized with DMSO and measured with the Synergy HTX multiplate reader (BiotTek, Bad Friedrichshall, Germany) at 550 nm. ANGIOGENIN levels in the culture medium were normalized to formazan formation of the pulp in the tooth slices.

### Reverse transcription quantitative polymerase chain reaction

One day after treatment with L-MIM or hypoxia, total RNA was isolated from DPC of both culture models as well as the pulp tissue of the tooth slices using the RNeasy Plus Mini Kit, according to the protocol of the manufacturer (Qiagen, Hilden, NW, Germany). We performed synthesis of cDNA with the High Capacity cDNA Reverse Transcription Kit (Applied Biosystems, Carlsbad, CA) and diluted for reverse transcription quantitative polymerase chain reaction (RT-qPCR) as recommended by the manufacturer. cDNA was amplified with TaqMan® Real-Time PCR Master Mix (Applied Biosystems) and TaqMan® assays (Applied Biosystems) for *angiogenin. Gapdh* served as housekeeping gene (Table 1). mRNA levels were calculated by the ΔΔCt method, relative to the *gapdh* gene expression levels.

**Table 1:** All TaqMan® assays that were used for qPCR are listed in this table ( Thermo Fisher Scientific, MA, USA)

Gene symbol	Gene name	Assay ID
*Ang*	*angiogenin*	Hs04195574_sH
*Gapdh*	*glyceraldehyde-3-phosphate dehydrogenase*	Hs02758991_g1

### Immunoassays

Supernatants of the monolayer, spheroid, and tooth slice organ cultures were assessed using ELISA for ANGIOGENIN (human angiogenin DuoSet® ELISA kit R&D Systems Europe, Ltd. Abingdon, UK). The absorbance was measured at 450 nm (Correction at 540 nm) in a Synergy HTX multiplate reader (BiotTek). The concentration of total ANGIOGENIN was calculated with the standard curve method following the protocol of the manufacturer.

### Statistical analysis

Statistical analysis was performed with IBM SPSS Statistics Version 23 (IBM Corporation, Armonk, NY, USA), using the Kruskal-Wallis-test *post hoc* Mann-Whitney-test corrected for multiple testing by Bonferroni correction. The level of significance was set at *p* < 0.05.

## Results

### L-mimosine and hypoxia increase the production of *angiogenin* / ANGIOGENIN in monolayer cultures of dental pulp-derived cells

**Fig. 1:**
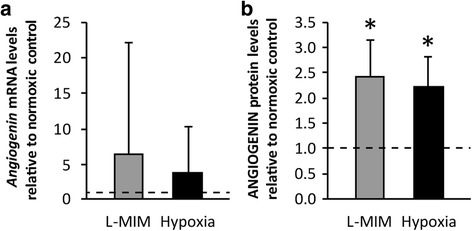
L-mimosine and hypoxia stimulate *angiogenin* / ANGIOGENIN production in monolayer cultures of dental pulp-derived cells. Dental pulp-derived cells (DPC) were incubated with L-mimosine (L-MIM) at 1 mM or hypoxia for 24 h in monolayer cultures. mRNA of *angiogenin* (**a**) and protein of ANGIOGENIN (**b**) were assessed by RT-qPCR and ELISA, respectively. **a** *Bars* represent mRNA levels as mean + standard deviation, relative to the normoxic control (*dashed line*). Two independent experiments were performed with three donors. Kruskal Wallis test *p* < 0.05. post hoc Mann-Whitney * *p* < 0.05 **b** *Bars* show mean + standard deviation of ANGIOGENIN relative to the normoxic control (*dashed line*). Two independent experiments were performed with three donors. Kruskal Wallis test *p* < 0.05. post hoc Mann-Whitney * *p* < 0.05

We measured the production of *angiogenin* at the mRNA level in response to L-MIM and hypoxia. *Angiogenin* was expressed in monolayer cultures of DPC under normoxic conditions. Modulation of *angiogenin* by both, L-MIM and hypoxia did not reach the level of significance (*p* < 0.05) in DPC monolayer cultures at the mRNA level (Fig. 1 a). At the protein level L-MIM and hypoxia increased ANGIOGENIN compared to the untreated cells (*p* < 0.05) as observed in the culture supernatants (Fig. 1 b). Our results show that hypoxia mimetic agents and hypoxia can increase the release of ANGIOGENIN in DPC cultured in monolayers.

### Echinomycin reduces the increase of *angiogenin* / ANGIOGENIN in response to L-mimosine and hypoxia

**Fig. 2:**
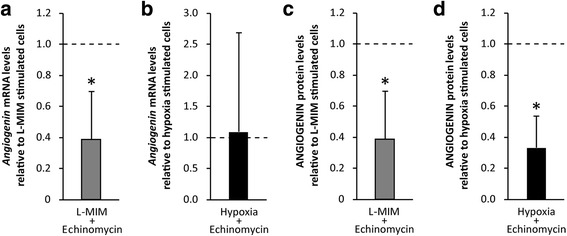
Hypoxia inducible factor-1 signaling is involved in the increase of *angiogenin* / ANGIOGENIN upon stimulation with L-mimosine or hypoxia. Dental pulp-derived cells (DPC) in monolayer cultures were treated with L-mimosine (L-MIM) at 1 mM or hypoxia with and without echinomycin at 1 μM to for 24 h. mRNA levels of *angiogenin* (**a, b**) and protein levels of ANGIOGENIN (**c, d**) were assessed by RT-qPCR and ELISA, respectively. **a, b** *Bars* represent mRNA levels as mean + standard deviation, relative to the normoxic control (*dashed line*). Experiments were conducted twice with three different donors, respectively. Kruskal Wallis test *p* < 0.05. post hoc Mann-Whitney * *p* < 0.05 **c, d** *Bars* represent mean + standard deviation of ANGIOGENIN relative to the normoxic control (*dashed line*). Experiments were conducted twice with 3 different donors, respectively. Kruskal Wallis test *p* < 0.05. post hoc Mann-Whitney * *p* < 0.05

To assess the role of HIF-1α in the underlying mechanism, inhibitor studies with echinomycin, an inhibitor of HIF-1 function, were performed. Echinomycin reduced mRNA production of *angiogenin* in the presence of L-MIM (*p* < 0.05) (Fig. 2 a, b). Furthermore, our data show that echinomycin reduces the effect of L-MIM and hypoxia on ANGIOGENIN protein production in DPC monolayer cultures (*p* < 0.05) (Fig. 2 c, d). These data demonstrate that HIF-1 activity is required for the effect of hypoxia mimetic agents and hypoxia on ANGIOGENIN production in monolayer cultures of DPC.

### L-mimosine, but not hypoxia, stimulates the production of *angiogenin* / ANGIOGENIN in spheroid cultures of dental pulp-derived cells

**Fig. 3:**
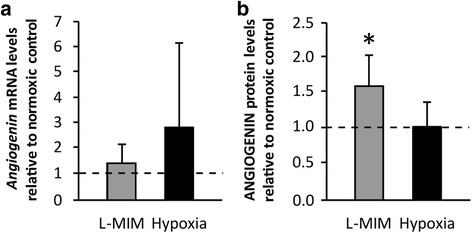
*Angiogenin* / ANGIOGENIN production in spheroid cultures of dental pulp-derived cells in response to L-mimosine and hypoxia. Dental pulp-derived cells (DPC) in spheroid cultures were treated with L-mimosine (L-MIM) at 1 mM or hypoxia for 24 h. mRNA levels of *angiogenin* (**a**) and protein levels of ANGIOGENIN (**b**) were assessed by RT-qPCR and ELISA, respectively. **a** *Bars* represent mRNA levels as mean + standard deviation, relative to the normoxic control (*dashed line*). Experiments were conducted twice with 3 different donors, respectively. Kruskal Wallis test *p* > 0.05. **b** *Bars* represent mean + standard deviation of ANGIOGENIN protein levels relative to the normoxic control (*dashed line*). Experiments were conducted twice with three different donors, respectively. Kruskal Wallis test *p* < 0.05. *post hoc* Mann-Whitney * *p* < 0.05 vs. control (*dashed line*)

To mimic the 3D matrix of the dental pulp we used the spheroid culture model. In these cultures, the increase of *angiogenin* mRNA upon L-MIM treatment did not reach the level of significance (*p* > 0.05) (Fig. 3 a). At the protein level ANGIOGENIN was increased by L-MIM (Fig. 3 b). The effect of hypoxia in the spheroid culture model did not reach the level of significance at both the mRNA and the protein level (Fig. 3 a, b). These suggest that the effect of hypoxia mimetic agents and hypoxia on ANGIOGENIN production are less pronounced under 3D than under 2D conditions.

### *Angiogenin* / ANGIOGENIN is produced in tooth slice organ cultures

**Fig. 4:**
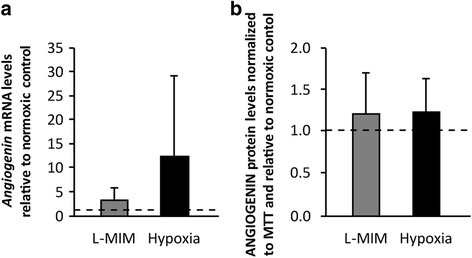
*Angiogenin* / ANGIOGENIN is produced in tooth slice organ cultures. Tooth slices were cultured with and without L-mimosine (L-MIM) or hypoxia. mRNA levels of *angiogenin* (**a**) and protein levels of ANGIOGENIN (**b**) were assessed by RT-qPCR and ELISA, respectively. **a** *Bars* represent mRNA levels as mean + standard deviation, relative to the normoxic control from tooth slices of two donors. **b** *Bars* represent mean + standard deviation of ANGIOGENIN protein levels normalized to MTT and relative to the normoxic control (*dashed line*) from tooth slices of two donors

Next we aimed to reveal the impact of hypoxia mimetic agents and hypoxia on the production of ANGIOGENIN in the dental pulp. Therefore, we applied the tooth slice organ culture model. Our results show that *angiogenin* mRNA was expressed in pulp tissue under normoxia, hypoxia, and in the presence of L-MIM (Fig. 4 a). We found a trend to an increase in *angiogenin* upon treatment with L-MIM and hypoxia. Due to the limited number of tooth slices, no statistical evaluation of *angiogenin* mRNA was performed as the resulting power was too low. Interestingly, no trend for a modulation of ANGIOGENIN protein levels in the culture supernatant was observed upon treatment with L-MIM or hypoxia (Fig. 4 b). Again no statistical evaluation of ANGIOGENIN protein levels was performed as the resulting power was too low due to the limited number of tooth slices. Overall these data show that ANGIOGENIN is produced in the dental pulp, also in the presence of hypoxia mimetic agents and hypoxia.

## Discussion

Angiogenin (*angiogenin* / ANGIOGENIN) is a key molecule in angiogenesis and healing. Here we show that ANGIOGENIN is increased in monolayer cultures of DPC in response to treatment with the hypoxia mimetic agent L-MIM and hypoxia. This increase in ANGIOGENIN was abolished in the presence of the HIF-1 activity inhibitor echinomycin suggesting that active HIF-1 is required for the increase in ANGIOGENIN. ANGIOGENIN was also produced in 3D spheroid cultures and in tooth slice organ cultures. However, the response of the cultures to treatment with L-mimosine and hypoxia depends on the culture model.

Here we present novel evidence showing the regulation of ANGIOGENIN in DPC. The hypoxia mimetic agent L-MIM and hypoxic conditions stimulate ANGIOGENIN in monolayer cultures of DPC. These results are in line with previous studies on other cell lines of non-oral origin such as retinal pigment endothelial cells or human renal proximal tubular epithelial cells in culture [31, 32].

Here we used DPC in monolayer cultures which represent a heterogeneous population and which can include cells, positive for mesenchymal stem cell markers, as well as low levels of hematopoetic stem cell markers [36–39]. In this setup it is unclear which population contributes to which extent to the observer effect. However, this setup closer represents the situation in the dental pulp than in isolated cell populations based on surface markers. To mimic the 3D structure of the pulp and therefore more closely the in vivo situation, we used 3D spheroid cultures of DPC in addition of 2D cultures [40]. Being aware of the background of a highly complex in vivo situation in the pulp-dentin complex which combines soft tissue containing fibroblasts, stem cells, blood vessels embedded in hard tissue, we therefore used the tooth slice organ culture [35]. Thereby we give a broad perspective on the effect of hypoxia mimetic agents and hypoxia on the production of *angiogenin* / ANGIOGENIN in the DPC. Our results show a differential response of *angiogenin* / ANGIOGENIN. It is therefore possible that cells cultured in the different 3D in vitro models are less sensitive to treatment with L-mimosine or hypoxia than cells cultured in monolayer cultures. It is also possible that cells in the core of the spheroids and within the pulp tissue of the tooth slices have already reached low levels of oxygen. Further treatment with hypoxia or L-MIM might therefore not be as effective. However, comparing the response at mRNA levels with the response at protein levels in particular in the tooth slice model suggests that *angiogenin* / ANGIOGENIN is produced in response to L-MIM and hypoxia, but is not released in its full extent. A clear limitation of this study is that the timing for sample collection for the evaluation of mRNA and protein was limited to one time point. Based on previous studies a stimulation period of 24 h was chosen for monolayer and spheroid cultures and 48 h was chosen for tooth slice cultures which showed a hypoxia-induced cellular response [24, 33, 35]. Both 24 and 48 h have been shown to be within the time frame of the effect of hypoxia on angiogenin production in non-oral cells [41]. However, it might be possible that the peak of *angiogenin* expression and ANGIOGENIN protein production is not at the same time point. Thus the kinetics of *angiogenin* / ANGIOGENIN production remains to be determined in future studies.

Hypoxia-based strategies are a promising approach in regenerative endodontics. Several studies have shown that targeting the cellular oxygen sensors with hypoxia mimetic agents or by hypoxic conditioning can support the pro-angiogenic capacity of the cells and increase cell survival and grafting capacity [24–30]. Also cell-free approaches for regenerative medicine have been described using conditioned medium, also known as secretome of cells of the dental pulp for tissue regeneration [42, 43]. Hypoxic pre-conditioning can support the pro-angiogenic capacity [43–45]. Here, in the present study we are the first to present that hypoxia mimetic agents and hypoxia can stimulate the production of ANGIOGENIN in DPC and that the effect depends on the in vitro model. It is possible that the elevated ANGIOGENIN levels contribute to the high pro-angiogenic capacity of secretome for cells of the dental pulp. However, due to the low stability of angiogenin it is unclear to which extent ANGIOGENIN might play a role in the therapeutic application of the secretome. While also strategies with recombinant ANGIOGENIN have been reported, a key factor seems to be the controlled release of ANGIOGENIN at the defect site [3, 4]. This challenge has stimulated research in ANGIOGENIN releasing scaffolds in particular for bone regenerative applications. Another strategy is the gene therapy approach [3, 4, 46]. Adeno-associated virus-mediated *angiogenin* gene transfer showed promising results [46]. However, no angiogenin-based strategy for regenerative endodontics was reported yet and the role of angiogenin in the dental pulp remains an enigma. What we have contributed are insights into the way how hypoxia mimetic agents and hypoxia regulate the production of angiogenin.

## Conclusions

In conclusion, our results show that the hypoxia mimetic agent L-MIM and hypoxia can stimulate angiogenin production in DPC and that this cellular response depends on HIF-1 activity. These results on the modulation of angiogenin in the dental pulp will be a primer for future studies which will address the role of angiogenin in pulp regeneration. Thus, the results of this study are of high relevance for pre-conditioning approaches in cell therapy and tissue engineering for regenerative endodontics.

## Abbreviations

ANG: Angiogenin
ANOVA: Analysis of variance
DPC: Dental pulp-derived cells
ELISA: Enzyme-linked immunosorbent assay
HIF: Hypoxia-inducible factor
L-MIM: L-mimosine
MTT: 3-(4,5-dimethylthiazol-2-yl)-2,5-diphenyltetrazolium bromide
qPCR: Quantitative polymerase chain reaction
RT: Reverse transcription
VEGF: Vascular endothelial growth factor
α-MEM: α-minimal essential medium
